# Evaluating compliance with the care standard of proactively assessing bone health in patients with diabetes: a pilot audit of practice across Asia by the Asia Pacific Consortium on Osteoporosis (APCO)

**DOI:** 10.1007/s11657-024-01399-y

**Published:** 2024-06-11

**Authors:** M. Chandran, N. Aftab, A. Amin, T. Amphansap, S. K. Bhadada, M. Chadha, D. C. Chan, F. L. Hew, S. Kaur, A. H. Khan, A. K. Kwee, L. T. Ho-Pham, S. Lekamwasam, D. C. Minh, A. Prasanth, R. Sharma, T. Valleenukul, N. Zehra, A. Mithal

**Affiliations:** 1 Osteoporosis and Bone Metabolism Unit, Department of Endocrinology, Singapore General Hospital, Singapore, Singapore; 2 Duke–NUS Medical School, Singapore, Singapore; 3 Aga Khan University Hospital, Karachi, Pakistan; 4 Osteoporosis and Geriatric Excellence Center, Department of Orthopaedics, Police General Hospital, Bangkok, Thailand; 5 Department of Endocrinology, Postgraduate Institute of Medical Education and Research, Chandigarh, India; 6 Department of Endocrinology, P. D. Hinduja Hospital & Medical Research Centre, Mumbai, India; 7 Department of Geriatrics and Gerontology, National Taiwan University Hospital, Taipei, Taiwan; 8 Department of Internal Medicine, College of Medicine, National Taiwan University, Taipei, Taiwan; 9 Subang Jaya Medical Centre, Subang Jaya, Selangor Malaysia; 10 Asia Pacific Consortium On Osteoporosis HK Ltd., Kwai Chung, Hong Kong People’s Republic of China; 11 Section of Chemical Pathology, Department of Pathology and Laboratory Medicine, Aga Khan University, Karachi, Pakistan; 12 BioMedical Research Center, Pham Ngoc Thach University of Medicine, Ho Chi Minh City, Vietnam; 13 Clinical Genetics Research Group, Saigon Precision Medicine Research Center, Ho Chi Minh City, Vietnam; 14 Department of Medicine, University of Ruhuna, Matara, Sri Lanka; 15 Department of Internal Medicine, Pham Ngoc Thach University of Medicine, Ho Chi Minh City, Vietnam; 16 Institute of Endocrinology and Diabetes, Max Healthcare, New Delhi, India; 17 Department of Orthopaedic Surgery, Bhumibol Adulyadej Hospital, Bangkok, Thailand

**Keywords:** Osteoporosis, Diabetes, Bone health, Asia, Fracture, Assessment, APCO

## Abstract

**Summary:**

This pilot audit explored how bone health is assessed patients with diabetes in diverse centres across Asia. Only 343 of 1092 (31%) audited patients had a bone health assessment, 27% of whom were diagnosed with osteoporosis. Quality improvement strategies are needed to address gaps in patient care in this area.

**Purpose:**

The Asia Pacific Consortium on Osteoporosis (APCO) Framework outlines clinical standards for assessing and managing osteoporosis. A pilot audit evaluated adherence to clinical standard 4, which states that bone health should be assessed in patients with conditions associated with bone loss and/or increased fracture risk; this report summarises the audit findings in patients with diabetes. A secondary aim was to assess the practicality and real-world use of the APCO bone health audit tool kit.

**Methods:**

Eight centres across Asia participated in the pilot audit, selecting diabetes as the target group. Participants reviewed their practice records for at least 20 consecutively treated patients with the target condition. Questions covered routine investigations, bone health assessment, osteoporosis diagnosis, and patient referral pathways. Data were summarised descriptively.

**Results:**

The participants represented public hospitals, university medical centres, and private clinics from India, Malaysia, Pakistan, Singapore, Taiwan, and Vietnam that see an estimated total of 95,000 patients with diabetes per year. Overall, only 343 of 1092 audited patients (31%) had a bone health assessment. Osteoporosis was subsequently diagnosed in 92 of 343 (27%) patients.

**Conclusion:**

Bone health was not assessed in most patients with diabetes. The results provide insight into current practices across diverse Asian centres and demonstrate the practical value of the audit tool kit. Participant feedback has been used to improve the tool kit. Results of this pilot audit are being used in the respective centres to inform quality improvement projects needed to overcome the gap in patient care.

**Supplementary Information:**

The online version contains supplementary material available at 10.1007/s11657-024-01399-y.

## Introduction

A steep increase in osteoporotic fractures is projected in the Asia–Pacific (AP) region due to a rapidly ageing population, together with urbanisation and increasingly sedentary lifestyles. Despite the enormous human and societal burden of fragility fractures, osteoporosis and its associated fractures is not widely recognised in the region as a major public health concern.

The Asia Pacific Consortium on Osteoporosis (APCO; https://apcobonehealth.org) is an independent organisation established in 2019 with a vision to reduce the burden of osteoporosis and fragility fractures in the Asia Pacific; the membership comprises osteoporosis experts from across the region [1]. The AP region is vast, highly populated, and culturally and socio-economically diverse. Accordingly, clinical practice guidelines for osteoporosis in the AP region show considerable variation across key areas. To address these issues, APCO developed a framework outlining the minimum clinical standards for assessing and managing osteoporosis, with the aim of informing future guidelines and improving osteoporosis care. Developed using a Delphi consensus approach, the 16 clinical standards are intended to promote evidence-based best practice whilst remaining pragmatic and adaptable to local conditions, thereby enabling the development of guidelines that are specific to individual countries [2].

As part of a ‘bottom-up’ approach to improving osteoporosis care in the region, APCO subsequently developed a bone health audit and quality improvement (QI) tool kit that front-line healthcare providers can use to benchmark their clinical practice against the relevant standard [3]. When APCO members were polled to ascertain which standard of care within the APCO Framework they wanted most a pilot audit to be conducted on, 75% voted for clinical standard 4, which states that individuals who have conditions associated with bone loss and/or increased fracture risk should be proactively identified to undergo assessment of bone health. A pilot audit was therefore conducted to assess current levels of adherence to this standard.

Several conditions, disease states and medications are associated with bone loss and an increased risk of fractures [4, 5], including diabetes, a highly prevalent disease associated with an increased risk of fractures at all levels of BMD [6, 7]. In 2021, diabetes affected an estimated 1 in 11 adults in Southeast Asia and 1 in 8 adults in the Western Pacific, according to the International Diabetes Federation [8]. The burgeoning case numbers are, as with osteoporosis, linked to ageing and the lifestyle changes associated with rapid urbanisation in developing areas, although the rise is not uniformly observed across the AP region [9]. Ethnicity is also a factor; in Singapore, diabetes is more common in Indian and Malay compared with Chinese individuals [10], and similar variation is reported amongst Asian subgroups in the USA [11]. Diabetes is associated with an increased risk of hip and other fractures; type 1 diabetes confers a higher risk, whilst the risk of hip fracture increases with the duration of type 2 diabetes [12, 13]. The cause of the increased fracture risk in diabetic individuals is multifactorial. In addition to an elevated falls risk, due to diabetic complications (e.g., vision impairment and poor balance due to neuropathy and sarcopenia) and treatment-induced hypoglycaemic episodes, hyperglycaemia also leads to bone fragility through complex and incompletely understood pathophysiological processes [14]. Diabetes was chosen as the target condition for the pilot audit given the substantial disease burden, and because it was felt that this would be a first step in addressing the significant care gap that likely existed in this area,

The question we sought to answer was whether bone health was being assessed in patients presenting for diabetes management in medical practices across the AP region. The primary objective of the pilot audit was to assess the care gaps in bone health management in patients with diabetes. A secondary aim was to evaluate the practicality and real-world use of the tool kit.

## Methods

Eight APCO clinician members volunteered to conduct the audit in their respective centres, all of which were medical clinics treating patients with both diabetes and osteoporosis. The participating clinicians in this pilot audit were provided with the APCO audit tool kit to refresh their knowledge of the clinical standards, and subsequently with the audit questionnaire pertaining to clinical standard 4, which is as follows: “Men and women who have conditions associated with bone loss and/or increased fracture risk should be proactively identified to undergo assessment of bone health.” [2]. The audit questionnaire was designed by a core committee within APCO and was distributed electronically in February 2023 to the participating clinicians, who completed the questionnaire based on a retrospective review of their patient records. No institutional review board approval was needed since this was a retrospective clinical audit in which data were anonymized before being submitted to APCO.

The audit incorporated 10 questions (Supplementary Table S1), that covered the clinic or specialist primarily responsible for managing patients in the target group; the referral pathway; estimated patient numbers managed over the past 12 months; investigations routinely performed; the number of patients who had a bone health assessment (and which tests or assessments were undertaken); the number of patients diagnosed with osteoporosis following a bone health assessment; and subsequent referral patterns.

Each clinician agreed to audit a consecutive series of a minimum of 20 patients with diabetes (all forms) most recently seen in their centre or clinical practice. The choice of 20 patients for the pilot audit was a pragmatic decision since it would be an easy number to achieve for clinicians, whilst providing adequate information on the application of the clinical standard and the usefulness of the audit questions. No maximum number of patients to be audited was imposed.

The anonymized data inputted into individual Excel spreadsheets by each participating clinician were then collated and analysed by the Project Manager and the Chairperson of APCO. Participants were separately asked to identify the barriers to assessing bone health in diabetic patients in their clinic. All data were summarised descriptively.

## Results

The eight participating centres included public hospitals, university medical centres, and private clinics, with three centres from India and one each from Malaysia, Pakistan, Singapore, Taiwan, and Vietnam. Two centres, in Malaysia and Taiwan, chose to audit all their patients treated over a 12-month period.

The primary responsibility of care for diabetic patients included in the audit rested with an endocrinologist in 7 of 8 centres and a geriatrician in one centre. Patients were referred by primary care physicians and specialists or were self-referred. Referring specialties included cardiology, nephrology, oncology, orthopaedics, geriatrics, gynaecology, rheumatology, and surgery. Five of 8 respondents also had patients referred via the emergency department.

All respondents routinely performed physical examinations, whilst 7 of 8 routinely performed blood tests and monitored lifestyle factors. Other routine investigations that were mentioned included eye and foot examinations, imaging using dual-energy x-ray absorptiometry (DXA) or lumbar spine x-ray, and ECG. Respondents also listed specific blood tests, including blood sugar/glycated haemoglobin (HbA1c), kidney and cholesterol panels, serum calcium, 25-hydroxy vitamin D, phosphorus, alkaline phosphatase, intact parathyroid hormone (iPTH), procollagen type 1 N propeptide (P1NP), and C-terminal crosslinking telopeptide of type 1 collagen (CTX-1).

Table 1 summarises the key findings of the pilot audit. The total number of audited patients in the diabetes target group was 1092, with a minimum of 20 from each centre. Across all centres, 343 of 1092 audited patients (31%) had a bone health assessment, representing 13% to 100% (median 34%) of the target group at each centre. Osteoporosis was diagnosed in 92 (27%) of the 343 patients who underwent bone health assessments. The percentage of patients diagnosed with osteoporosis following a bone health assessment was 15% to 88% (median 54%) across the participating centres.

**Table 1:** Results of audit on bone health assessment in patients with diabetes in Asian medical centres

Participating centres		Estimated diabetic patients managed over 12 months, *N*	Bone health assessment in audited patients		
Country	Description		Audited patients, *N*	Bone health assessment, *n* (%)^a^	Osteoporosis diagnosis, *n* (%)^b^
India, centre 1	Private multispecialty medical centre	3960	20	20 (100%)	10 (50%)
India, centre 2	Public University teaching hospital	25,000	20	7 (35%)	4 (57%)
India, centre 3	Private clinic	2500	20	10 (50%)	6 (60%)
Malaysia	Private clinic	875	875	238 (27%)	35 (15%)
Pakistan	Private University / academic medical centre	60,000	25	8 (32%)	7 (88%)
Singapore	Specialist clinic within public teaching hospital	2200	40	6 (15%)	4 (67%)
Taiwan	University teaching hospital	60	60	50 (83%)	25 (50%)
Vietnam	University teaching hospital	768	32	4 (13%)	1 (25%)
**Total**	–	**95,363**	**1092**	**343 (31%)**	**92 (27%)**

^a^Percentage of patients with diabetes; ^b^percentage of patients with bone health assessment

Table 2 summarises the main types of assessment performed in each centre for the subset of patients whose bone health was assessed. Overall, 305 of 343 patients (89%) had serum calcium / vitamin D measured, 148 (43%) had a DXA scan, and 97 (28%) had a fracture risk assessment (e.g., using Fracture Risk Assessment Calculator [FRAX®; https://frax.shef.ac.uk/FRAX/], Garvan Fracture Risk Calculator [https://www.garvan.org.au/research/bone-fracture-risk-calculator], or Osteoporosis Self-Assessment Tool for Asians [OSTA] [15]). Fracture risk assessment was not performed in the four centres based in India and Pakistan.

**Table 2:** Types of bone health assessment performed in patients with diabetes in Asian medical centres

Participating centre	Patients with any bone health assessment, *N*	Type of bone health assessment, *n* (%)		
		DXA scan	Serum calcium / vitamin D	Fracture risk assessment
India, centre 1	20	17 (85%)	17 (85%)	0
India, centre 2	7	7 (100%)	7 (100%)	0
India, centre 3	10	4 (40%)	6 (60%)	0
Malaysia	238	58 (24%)	217 (91%)	62 (26%)
Pakistan	8^a^	3 (38%)	2 (25%)	0
Singapore	6	6 (100%)	6 (100%)	6 (100%)
Taiwan	50	50 (100%)	50 (100%)	25 (50%)
Vietnam	4	4 (100%)	0	4 (100%)
**Total**	**343**	**149 (43%)**	**305 (89%)**	**97 (28%)**

*DXA* dual-energy x-ray absorptiometry

^a^Three patients underwent orthopantomography as their bone health assessment

Following a diagnosis of osteoporosis, six out of eight participating centres reported that the patients continued to be seen by the specialist respondent. However, in Pakistan (*n* = 7) and Taiwan (*n* = 25), all patients diagnosed with osteoporosis were referred to primary care physicians or other specialists/bone health experts, in addition to ongoing management by the responding clinician. In Taiwan, treatment for osteoporosis was prescribed or initiated before referral to a fracture liaison service (FLS).

## Discussion

Globally, the number of individuals aged over 50 years who are at high risk of osteoporotic fracture is projected to double between 2010 and 2040, with the majority (55%) of high-risk individuals living in Asia [16]. A regional audit conducted by the International Osteoporosis Foundation (IOF) in 2013 highlighted some of the challenges facing the diverse AP region: ageing populations, high and increasing fracture rates, widespread low calcium intake and vitamin D deficiency, inadequate epidemiological data, and barriers to diagnosis and treatment resulting from a lack of prioritisation of osteoporosis, inadequate DXA coverage in many areas, and lack of reimbursement [17]. A systematic review of data from seven developed AP economies found the osteoporosis prevalence to be 10–30% in women aged over 40 years and up to 10% amongst men, with hip fracture rates of 500–1000 per 100,000 person-years in those aged over 50 years [18].

Whilst there are shared challenges across the region, the countries participating in the pilot audit have differing contexts for managing osteoporosis. Singapore is well resourced, and osteoporosis is designated a national health priority. A successful FLS is now integrated into the operating budget of public hospitals and polyclinics, and patients can access their national health insurance to pay for osteoporosis clinic visits [19]. Easy-to-read guidelines for primary care are widely used in Singapore, with primary practitioners identifying the limited availability of anti-osteoporosis medications at their practices as the leading barrier to managing osteoporosis [20, 21]. By contrast, in Pakistan, osteoporosis is not prioritised, awareness remains low, and osteoporosis frequently goes undiagnosed and untreated in patients with osteoporotic hip fractures [22, 23]. Factors contributing to suboptimal management include the involvement of multiple specialties and a lack of clinical guidelines [22]. In India, osteoporotic fractures occur at a younger age than in Western populations, but individual awareness is poor and clinicians may also lack relevant knowledge [24, 25]. Access to DXA remains limited outside major centres in India and Sri Lanka. In Malaysia, primary care doctors identified inability to access bone mineral densitometry as the leading barrier to osteoporosis screening and management (90.6%) [26]. Applying country-specific FRAX® intervention thresholds has the potential to optimise DXA referrals and guide treatment initiation in areas with resource constraints [27, 28]. In Thailand, guidelines recommend using the Thai-specific FRAX® tool, whilst noting the paucity of data on major non-hip fractures and the need to establish appropriate intervention thresholds [29]. In the absence of country-specific FRAX® tools for Malaysia or Vietnam, Malaysian guidelines recommend using the appropriate ethnic-specific algorithm from the Singapore or Hong Kong FRAX® tools [30, 31], whereas simple predictive models have been trialled in Vietnam to identify individuals at high risk of osteoporosis [32]. Although Vietnam recognises osteoporosis as a major public health issue, most patients remain undiagnosed and untreated due to resourcing and infrastructure deficits [33]. In Taiwan, where guidelines were updated in accordance with the APCO Framework, FLSs have been shown to improve patient outcomes, but lack of reimbursement of anti-osteoporosis medications for patients with confirmed osteoporosis, but no associated fracture, remains a barrier to care [34, 35].

A review of osteoporosis guidelines from the AP region during the development of the APCO Framework did indicate a growing awareness, at least amongst specialists, of diabetes as a risk factor for osteoporotic fracture [2]. However, lack of awareness of fracture risk amongst patients with diabetes remains of concern globally. In Canada, most outpatients aged ≥ 50 years did not believe that diabetes increased their risk of fracture (81%) or falls (68%), and only 9% said their doctor had informed them of the risk of diabetes-related fracture, even though 15% reported a diagnosis of osteoporosis, 14% had experienced a fragility fracture after the age of 40, and 27% had fallen in the previous 6 months [36]. In Malaysia, only one-third of patients with diabetes had a high level of osteoporosis knowledge [37], whilst in hospitalised, predominantly elderly women in Singapore, inadequate awareness of osteoporosis was significantly more common amongst diabetic patients than in those without diabetes [38]. This lack of awareness suggests a missed opportunity for patient education and interventions to prevent debilitating fractures, and indeed only half of AP country-specific osteoporosis guidelines published from 2015 recommend informing patients about the link between diabetes and fracture risk [2].

Most participants in this pilot audit were endocrinologists, but primary healthcare practitioners are also well placed to engage individuals with diabetes in their own care by providing information on calcium and vitamin D intake, sun exposure, exercise, and the relationship between osteoporosis and fracture risk (clinical standard 8 of the APCO Framework) [2].

In the pilot audit, only 31% of all 1092 audited patients with diabetes underwent a bone health assessment. Osteoporosis was subsequently diagnosed in a median of 54% of patients. The number of patients with diabetes treated at each centre over a 12-month period varied widely (Table 1).

The fact that more than two-thirds of patients with diabetes in the audit had no bone health assessment was surprising, given that the participating clinicians were all osteoporosis experts, but likely reflects the reality of busy practices with time and resource constraints. One centre in India had a 100% bone health assessment rate because the participating clinician had mandated this assessment for all patients with diabetes, and two other centres had an assessment rate of 50% or higher. Participants reported various barriers to assessing bone health in diabetic patients, including: a lack of awareness by both clinicians and patients alike of the importance of bone health; osteoporosis being considered a lower priority than other, more immediately pressing chronic diseases; the belief that other diabetic complications were more critical; resistance to additional testing in asymptomatic patients; time constraints; the cost of investigations such as DXA; limited availability of investigations outside major centres; and limited capacity for follow-up visits. The 2013 IOF osteoporosis audit recorded variable diagnostic costs and reimbursement policies across the AP region as potential barriers to care [17].

Regarding the type of bone health assessment performed, only the Singaporean centre consistently applied DXA, serum calcium/vitamin D assessment and fracture risk assessment (Table 2). Assessing fracture risk in the context of diabetes is not straightforward, especially in type 2 diabetes, in which patients may have normal BMD with an elevated fracture risk, and in Asian countries, where the use of standard thresholds derived from Caucasian populations may be inappropriate [39]. Fracture risk assessment tools are designed to identify patients at risk regardless of BMD, but both BMD and FRAX® consistently underestimate risk in patients with diabetes [7, 39]. None of the participating centres from India and Pakistan used fracture risk assessments during the audit, although two Indian centres indicated they would expect to assess fracture risk in 20–22% of patients over a 12-month period. Clinical standard 5 of the APCO Framework recommends routine use of country-specific (if available) fracture risk assessment or osteoporosis screening tools [2].

The limitations of the current study primarily relate to its intended function as a pilot audit involving a small group of motivated and often highly experienced clinicians who were interested in participating. It is probable that a wider group of clinicians without a specialist interest in the area would be even less aware of, and less likely to adhere to, the clinical standards outlined in the APCO Framework. However, an advantage of the sample is the mix of hospitals and other clinical practices, providing real-world data on current practices across a range of settings.

## Conclusion

The primary goals of the pilot audit were to evaluate how patients with diabetes were being assessed with regard to their bone health in diverse centres in Asia, and to identify the care gaps and pathways in such management. We believe that this goal has been achieved through the audit which revealed an overwhelming gap in care, despite the fact that the majority of the participants in the audit were endocrinologists. There is an urgent need to address this gap. It is imperative that awareness about the importance of appropriate bone health care in diabetes be expanded amongst both healthcare practitioners and patients alike.

Initiating quality improvement projects through iterative Plan-Do-Study-Act cycles at individual centres using the findings from the pilot audit is the next step [40]. It is hoped that the lessons learned from the implementation of the audit at individual institutional level will be utilised to build momentum so that quality improvement measures and the clinical standard advocated in the APCO Framework will be adopted in national osteoporosis guidelines as well as be employed to lobby for policy changes at national and regional level.

The secondary aim of the audit was to assess the practicality and real-world use of the APCO QI tool kit, which is available on the APCO web site (https://apcobonehealth.org/apco-bone-health-qi-tool-kit/). The tool kit has indeed proved to be a practically useful instrument with the audit being able to be successfully conducted across varied centres in diverse parts of Asia. Based on feedback from the audit participants, clearer guidance on how to conduct the audit is now provided in the tool kit. In addition, the resources available within the tool kit now include a slide-deck explaining the clinical standard chosen, instructions on how to conduct the audit and utilise the results generated, a Word document that lists the audit questions, an Excel file that can be used to input the raw data obtained from the audited patients, and an automated calculator to generate results from the audit data. It is thus hoped that the APCO bone health tool kit will serve as an invaluable aid for healthcare providers around the world to conduct similar audits of their practices.

## Supplementary Information

Below is the link to the electronic supplementary material.

Supplementary file1 (DOCX 34 KB)
