# Supplementary material for: Evaluating compliance with the care standard of proactively assessing bone health in patients with diabetes: a pilot audit of practice across Asia by the Asia Pacific Consortium on Osteoporosis (APCO)
Source: Arch Osteoporos. 2024 Jun 11;19(1):48. doi: 10.1007/s11657-024-01399-y (PMC11166814; doi:10.1007/s11657-024-01399-y)
Supplement: Supplementary file 1 — Supplementary file1 (DOCX 34 KB) [file 11657_2024_1399_MOESM1_ESM.docx]

APCO Pathfinder Pilot Audit Questionnaire for Clinical Standard 4

| **The APCO Framework Clinical Standard 4^[[1]](#footnote-1)^** |
| --- |
| Men and women who have **conditions** associated with bone loss and/or increased fracture risk should be proactively identified to undergo assessment of bone health.  *Action at national level*: A commentary should be included in new or revised osteoporosis clinical guidelines to highlight common prevalent conditions in the country or region. |

## Audit Questions

1. **Who is your target patient group for the audit among those with conditions** **associated with bone loss and/or increased fracture risk?**

Patients with:

- Rheumatoid arthritis
- Malabsorption disorders
- Hyperthyroidism
- Diabetes
- Multiple myeloma
- Chronic obstructive pulmonary disease
- HIV
- Dementia
- Anorexia nervosa
- Early menopause
- Other specific target group (please specify): _________________________________________

1. **Who is primarily responsible for the patients in your target group?**

- Specialist physician Specify: ________________________ (e.g. rheumatologist, geriatrician)
- Outpatient clinic
- Other Specify: __________________________

*(Note: The purpose of this question is to identify the clinic/department to be audited).*

1. **How do the patients in your target group come to your medical centre/practice?**

- By referral from a primary care physician?
- By referral from a specialist physician? Specify: ____________ (e.g. gynaecologist)
- From the emergency department?
- Through direct patient contact?
- Other Specify: __________________________

1. **For the patients in your target group, estimate the total number of patients managed in your clinic/department in the past 12 months.**

Total number: _________________________________________

1. **For the patients in your target group, which investigations were routinely undertaken?**

- Blood tests (e.g. blood count, hormone levels, inflammatory markers)
- Physical examination
- Monitoring of lifestyle risk factors (e.g. smoking cessation)
- Other *[add all relevant investigations that are likely to be undertaken (e.g. imaging or neurological assessments, etc.)]*

1. **In the past 12 months, how many patients within the nominated clinic/department had a bone health assessment?**

Total number: _________________________________________

1. **Which tests or assessments were undertaken?**
   - How many underwent a DXA scan?
   - How many had serum calcium/vitamin D measurements?
   - How many had a fracture risk assessment (e.g. using FRAX, Garvan Fracture Risk Calculator or OSTA)?
2. **In the past 12 months, how many patients in your nominated clinic/department were diagnosed with osteoporosis following a bone health assessment?**

Total number: _________________________________________

1. **For the patients diagnosed with osteoporosis, where were they referred for their bone health management?**

- Specialist physician / bone health expert
  Specify: _________________________
- Primary care physician
- Other Specify: __________________________
- Lost to follow-up

1. **For the patients diagnosed with osteoporosis, how many patients (e.g. 10) were referred with these options? Please place a number for each option (even if zero):**

- Recommendations for treatment to the patient/carer only
- Recommendations for treatment to the referring healthcare professional
- Summary of results of bone health investigation
- Treatment prescribed/initiated before referral

1. Chandran M, et al. *Osteoporos Int* 2021; 32:1249–75. [↑](#footnote-ref-1)
